# Spatial Clustering of Suicide and Associated Community Characteristics, Idaho, 2010–2014

**DOI:** 10.5888/pcd16.180429

**Published:** 2019-03-28

**Authors:** Ahmed M. Kassem, Kris K. Carter, Christopher J. Johnson, Christine G. Hahn

**Affiliations:** 1 Epidemic Intelligence Service, Division of Scientific Education and Professional Development, Centers for Disease Control and Prevention, Atlanta, Georgia; 2 Division of Public Health, Idaho Department of Health and Welfare, Boise, Idaho; 3 Center for Preparedness and Response, Centers for Disease Control and Prevention, Atlanta, Georgia; 4 Cancer Data Registry of Idaho, Boise, Idaho

## Abstract

**Introduction:**

In 2015, Idaho had the fifth highest suicide rate in the United States. Little is known about the characteristics of areas in Idaho with high suicide rates. To aid suicide prevention efforts in the state, we sought to identify and characterize spatial clusters of suicide.

**Methods:**

We obtained population data from the 2010 US Census and the 2010–2014 American Community Survey, analyzed data on suicides from death certificates, and used a discrete Poisson model in SaTScan to identify spatial clusters of suicide. We used logistic regression to examine associations between suicide clustering and population characteristics.

**Results:**

We found 2 clusters of suicide during 2010–2014 that accounted for 70 (4.7%) of 1,501 suicides in Idaho. Areas within clusters were positively associated with the following population characteristics: median age ≤31.1 years versus >31.1 years (multivariable-adjusted odds ratio [aOR] = 2.4; 95% confidence interval [CI], 1.04–5.6), >53% female vs ≤53% female (aOR = 2.7; 95% CI, 1.3–5.8; *P* = .01), >1% American Indian/Alaska Native vs ≤1% American Indian/Alaska Native (aOR = 2.9; 95% CI, 1.4–6.3), and >30% never married vs ≤30% never married (aOR = 3.4; 95% CI, 1.5–8.0; *P* = .004).

**Conclusion:**

Idaho suicide prevention programs should consider using results to target prevention efforts to communities with disproportionately high suicide rates.

**Summary**

**What is already known on this topic?**

A few studies in the southeastern United States have identified spatial clusters of suicide at the county and census tract levels.

**What is added by this report?**

This study identified spatial clusters of suicide at the census block group level in Idaho, a northwestern rural state.

**What are the implications for public health practice?**

Because all of Idaho is federally designated as having a shortage of mental health providers, this study will inform stakeholders targeting Idaho communities with disproportionately high suicide rates at a more detailed level.

## Introduction

In the United States, suicide is the tenth leading cause of death; more than 44,000 suicides were reported in 2015 (1). From 2000 to 2015, the US age-adjusted suicide rate increased by 28%, from 10.4 per 100,000 population to 13.3 per 100,000 population (2). Suicide results in substantial medical and work-loss costs; lifetime costs were estimated to exceed $56 billion in 2015 (1); this conservative estimate did not account for underreporting of suicides and other societal costs (eg, pain and suffering, justice system). Beyond the economic burden, suicide negatively affects families and community members, who may have long-lasting mental health problems and other life-changing difficulties (3).

Suicide rates vary in the United States by geographic location. During 2011–2015, the age-adjusted suicide rate was higher in the West than in the Northeast (14.0 per 100,000 population [West census region] vs 9.8 per 100,000 population [Northeast census region]) (1). Although suicide rates increased across all levels of urbanization in the United States during 1999–2015, rates were higher in less urban areas than in more urban areas (4). Because geographic differences are not fully explained by demographic patterns (5), they could be attributed to other factors, such as lack of access or poor access to quality mental health care, low socioeconomic status, and weak social cohesion in areas with high suicide rates (6–8). Increased access to lethal means could be another explanatory factor in areas with higher suicide rates (9).

A comprehensive public health approach to suicide prevention, in contrast to an approach that focuses on mental health treatment, can address multiple risk factors across the lifespan (10). Although a public health suicide prevention approach is warranted in communities nationwide (10), it is essential to focus on communities with disproportionately high suicide rates to eliminate geographic disparities and reduce suicide altogether (11,12). Furthermore, examination of suicide data at a fine-scale geographic level is needed to identify these communities for efficient planning and targeting effective prevention efforts, especially when resources are limited. 

Several types of suicide clusters have been reported, including mass clusters, space–time clusters, and spatial clusters (13). Spatial cluster analysis has been used to identify communities with disproportionately high suicide rates, because spatial cluster analysis overcomes the “small numbers problem” (in which rates for areas with small populations have wider variability and less reliability than rates for areas with large populations) inherent in spatial analysis and allows for statistical assessment of rates across geographic units (14). A study in 2012 found 2 high-risk spatial clusters of suicide during 1999–2008 that comprised 15 of 120 counties in Kentucky (15). Another study, in 2017, found 24 high-risk spatial clusters of suicide during 2001–2010 that comprised 491 of 3,154 census tracts in Florida (16). Studies of suicide in Scotland, Australia, São Paulo, and Québec used the same methodology (17–20). To our knowledge, no study of suicide using spatial cluster analysis has been conducted in rural or western parts of the United States.

In Idaho, a northwestern rural state with a population of 1.7 million, suicide is a major public health problem (21). Idaho consistently ranks among the top 10 states with the highest suicide rates, with an age-adjusted suicide rate of 22.2 per 100,000 population, compared with 13.3 per 100,000 population nationally in 2015 (1). Eighteen of 44 counties in Idaho had an age-adjusted suicide rate of 22.0 per 100,000 population or more during 2010–2014 (21). However, these rates are likely unstable because of the small numbers problem (21). Because all of Idaho is federally designated as having a shortage of mental health providers (22), targeting Idaho communities with disproportionately high suicide rates at a more detailed level than the county level (because some counties are very large in area) is crucial. Therefore, we sought to identify and characterize areas with spatial clusters of suicide at the neighborhood level in Idaho. We examined whether there are geographic areas in Idaho that have statistically significant higher rates of suicide than expected, compared with other geographic areas in the state, and we explored their characteristics. For a complete representation of suicide in Idaho, we also described the epidemiology of residents who died by suicide.

## Methods

We used a retrospective ecological study design to investigate suicides among Idaho residents during 2010–2014. We did not include suicides occurring in Idaho among out-of-state persons, because an objective of our study was to examine the characteristics of communities in which Idaho residents who died by suicide lived at the time of death. We used the census block group as a proxy for neighborhood. A census block group is a statistical division of a census tract that covers a contiguous area and generally has a population size of 600 to 3,000 people, whereas a census tract is a relatively permanent statistical subdivision of a county and generally has a population size of 1,200 to 8,000 people (23). Our study was deemed nonresearch public health practice by the Idaho Division of Public Health’s Research Determination Committee.

We obtained individual-level data on suicides from death certificates stored by the Idaho Bureau of Vital Records and Health Statistics, and for the spatial cluster analysis, we aggregated data on suicides to census block group. Although some suicide reporting systems and research exclude suicides among persons younger than 10 years, we did not exclude any age group, in accordance with the standard practice in Idaho (21). We identified suicides by the established *International Classification of Diseases, Tenth Revision*, codes as follows: X60.0–X84.9, Y87.0, and U03.9 (24). Death certificates included information on sex, age, ethnicity, race, education, marital status, military status (based on the question “Ever in US Armed Forces?”), occupation, and mechanism of injury. We geocoded residential addresses from death certificates to obtain 15-digit census block group identifiers. We completed geocoding by using the Automated Geospatial Geocoding Interface Environment System (25). In total, 98.5% of residential addresses were matched to a census block group identifier; we excluded 23 suicides without a matched census block group identifier. We used census block group identifiers to merge suicide data with other data sources.

We obtained data on population estimates from the 2010 US Census and data on community characteristics from the 2010–2014 American Community Survey’s 5-year estimates (26). We measured the following community characteristics, suggested by previous studies (16–20), in proportions as appropriate: female; median age; American Indian or Alaska Native; Hispanic or Latino; persons never married; persons in single-parent families; persons with less than a high school education (ie, did not receive a regular high school diploma, GED, or alternative credential); unemployed persons, median household income; persons in poverty; persons in renter-occupied housing units; persons with disabilities; and persons with no health insurance. In addition to showing demographic patterns, these characteristics capture dimensions of social cohesion and economic deprivation that could be associated with suicide (16–20).

### Data analysis

Using information from death certificates, we first calculated descriptive statistics of residents who died by suicide and stratified these data by sex. We used the Pearson χ^2^ test for categorical variables (or Fisher exact test for <5 expected cell counts) and *t *tests for continuous variables. Next, we conducted spatial cluster analysis by using SaTScan version 9.4 (Martin Kulldorff and Information Management Services Inc), free software that uses scan statistics to identify clusters (27). We used the discrete Poisson model (28) to scan for nonoverlapping geographical areas (census block groups) with significantly high rates of suicide. In SaTScan, we used population size (default of 50% of the total population at risk) to specify the maximum spatial cluster size; circular spatial window shape, adjusted for sex and age distributions; and the default of 999 Monte Carlo replications. We selected the spatial clusters with *P* < .10 for the subsequent analyses.

We used logistic regression models to examine associations between community characteristics and suicide clustering. Suicide clustering was constructed as a binary outcome variable indicating whether a census block group belonged to a spatial cluster of suicide (with *P* < .10). To simplify interpretation and use of findings for a wider audience, we dichotomized each variable for community characteristics into high and low levels. Except for age and income, we constructed the variables to compare the highest quartile with the lowest 3 quartiles for each variable. For age and income, we constructed the variables to compare the lowest quartile with the highest 3 quartiles for each variable. We fit a series of univariable models to examine association of each community characteristic with suicide clustering. Community characteristics that were significant at *P* < .05 in the univariable models were included in a multivariable model to identify the most important community characteristics related to suicide clustering. We performed model diagnostics, including goodness of fit and multicollinearity assessments, which did not indicate problems. We used SAS version 9.3 (SAS Institute Inc) for all statistical analyses other than spatial cluster analysis, and we used ArcGIS version 10 (Environmental Systems Research Institute, Inc) for cartographic displays of spatial clusters.

## Results

During 2010–2014, 1,501 Idaho residents died by suicide. Most residents who died by suicide were male (78.5%), aged 35 to 64 years (53.7%), non-Hispanic (95.8%) and white (97.0%) (Table 1). Overall, male and female residents who died by suicide did not significantly differ by the demographic characteristics examined. However, they significantly differed by marital status, military status, occupational status, and suicide method. The proportion of divorced persons was higher among females (32.6%) than males (25.0%), and the proportion of persons never married was higher among males (32.9%) than females (27.0%). The proportion of those who served in the military was higher among males (26.8%) than among females (3.1%). The proportion of those who were homemakers and those who had never worked or were disabled was higher among females (19.7% and 5.0%, respectively) than males (0.2% and 2.7%, respectively). For mechanism of injury, males (67.6%) were more likely than females (34.4%) to die by a firearm, and females (36.2%) were more likely than males (11.1%) to die by poisoning.

**Table 1:** Characteristics of Residents Who Died by Suicide, Stratified by Sex, Idaho, 2010–2014^a^

Characteristic	Total^b^ (n = 1,501)	Male (n = 1,178)	Female (n = 323)	*P* Value^c^
**Age, mean (SD), y**	45.6 (18.7)	45.9 (19.4)	44.4 (16.3)	.15
**Age group, n (%), y **				
<15	20 (1.3)	18 (1.5)	2 (0.6)	.008
15–24	231 (15.4)	182 (15.5)	49 (15.2)	
25–34	212 (14.1)	171 (14.5)	41 (12.7)	
35–44	255 (17.0)	192 (16.3)	63 (19.5)	
45–54	303 (20.2)	226 (19.2)	77 (23.8)	
55–64	248 (16.5)	186 (15.8)	62 (19.2)	
65–74	117 (7.8)	99 (8.4)	18 (5.6)	
75–84	69 (4.6)	62 (5.3)	7 (2.2)	
≥85	46 (3.1)	42 (3.6)	4 (1.2)	
**Ethnicity, n (%)**				
Hispanic	63 (4.2)	47 (4.0)	16 (5.0)	.45
Non-Hispanic	1,437 (95.8)	1,130 (96.0)	307 (95.1)	
**Race, n (%)**				
White	1,456 (97.0)	1,146 (97.3)	310 (96.0)	.30
Black	4 (0.3)	4 (0.3)	0	
American Indian	24 (1.6)	15 (1.3)	9 (2.8)	
Asian Pacific Islander	8 (0.5)	6 (0.5)	2 (0.6)	
Other or mixed race	9 (0.6)	7 (0.6)	2 (0.6)	
**Education, n (%)**				
<High school^d^	264 (17.7)	210 (18.0)	54 (16.9)	.18
High school	607 (40.8)	488 (41.8)	119 (37.2)	
>High school	617 (41.5)	470 (40.2)	147 (45.9)	
**Marital status, n (%)**				
Married, including married but separated	537 (35.9)	423 (36.1)	114 (35.4)	.03
Widowed	87 (5.8)	71 (6.1)	16 (5.0)	
Divorced	398 (26.6)	293 (25.0)	105 (32.6)	
Never married	472 (31.6)	385 (32.9)	87 (27.0)	
**Military status, n (%)**				
Yes	323 (21.6)	313 (26.8)	10 (3.1)	<.001
No	1,170 (78.4)	857 (73.3)	313 (96.9)	
**Occupational status, n (%)**				
Student	127 (8.6)	97 (8.3)	30 (9.4)	<.001
Homemaker, housewife	65 (4.4)	2 (0.2)	63 (19.7)	
Never worked, disabled	47 (3.2)	31 (2.7)	16 (5.0)	
Other occupational groups	1,246 (83.9)	1,035 (88.8)	211 (65.9)	
**Mechanism of injury,^e^ n (%)**				
Poisoning (X60–X69)	248 (16.5)	131 (11.1)	117 (36.2)	<.001
Hanging, strangulation, suffocation, drowning and submersion (X70–X71)	294 (19.6)	216 (18.3)	78 (24.2)	
Firearm (X72–X74)	907 (60.4)	796 (67.6)	111 (34.4)	
Other methods (X75–X84, Y87)	52 (3.5)	35 (3.0)	17 (5.3)	

a Individual-level data on suicides obtained from death certificates stored by the Idaho Bureau of Vital Records and Health Statistics.

b The total number of participants for each variable varies because of missing values.

c Based on the Pearson χ^2^ test or Fisher exact test for categorical variables and *t* test for continuous variables.

d Did not receive a regular high school diploma, GED, or alternative credential.

e Based on *International Classification of Diseases, Tenth Revision* (24). No death using the U03.9 ICD-10 code was reported.

### Spatial clusters of census block groups with high suicide rates

SaTScan identified a “most likely” cluster and 9 secondary clusters (Table 2). The 2 identified spatial clusters (with *P* < .10) of census block groups with disproportionately high suicide rates during 2010–2014 accounted for 70 (4.7%) of 1,501 deaths by suicide (Figure). The “most likely” spatial cluster, comprising 25 census block groups and a population of 30,405, was found in southeastern Idaho. During 2010–2014, 54 suicides occurred in this spatial cluster, whereas 28 suicides were expected, indicating that the suicide rate was 90% higher inside the cluster than outside (relative risk = 1.9, *P* = .04). A secondary spatial cluster with *P* < .10 was identified in northeastern Idaho. This secondary spatial cluster, comprising 6 census block groups and a population of 4,391, had 16 suicides, whereas 4 suicides were expected. The suicide rate was more than 3 times higher inside this cluster than outside (relative risk = 3.6, *P* = .06).

**Table 2:** Spatial Clusters of Suicide by Residential Location, Idaho, 2010–2014^a^

Cluster No.	Cluster	No. of Census Block Groups	Population^b^	Observed No. of Suicide Deaths	Expected No. of Suicide Deaths	Annual Deaths per 100,000	Relative Risk	Log-Likelihood Ratio	*P*
1	Most likely	25	30,405	54	28.4	35.9	1.9	9.4	.04
2	Secondary	6	4,391	16	4.5	67.6	3.6	8.9	.06
3	Secondary	11	14,084	28	13.3	39.8	2.1	6.3	.55
4	Secondary	22	25,347	44	25.3	32.8	1.8	5.8	.69
5	Secondary	1	1,600–1,700	7	1.7	78.7	4.1	4.7	.95
6	Secondary	3	2,947	10	3.2	58.1	3.1	4.5	.97
7	Secondary	3	2,040	8	2.3	67.1	3.6	4.4	.98
8	Secondary	5	4,896	12	4.4	51.0	2.7	4.4	.98
9	Secondary	30	60,471	72	51.0	26.6	1.4	4.0	>.99
10	Secondary	1	500–600	4	0.6	117.9	6.3	4.0	>.99

a Individual-level data on suicides obtained from death certificates stored by the Idaho Bureau of Vital Records and Health Statistics. Data on population estimates obtained from the 2010 US Census and data on community characteristics from the 2010–2014 American Community Survey’s 5-year estimates (26).

b Adjusted for sex and age.

**Figure:**
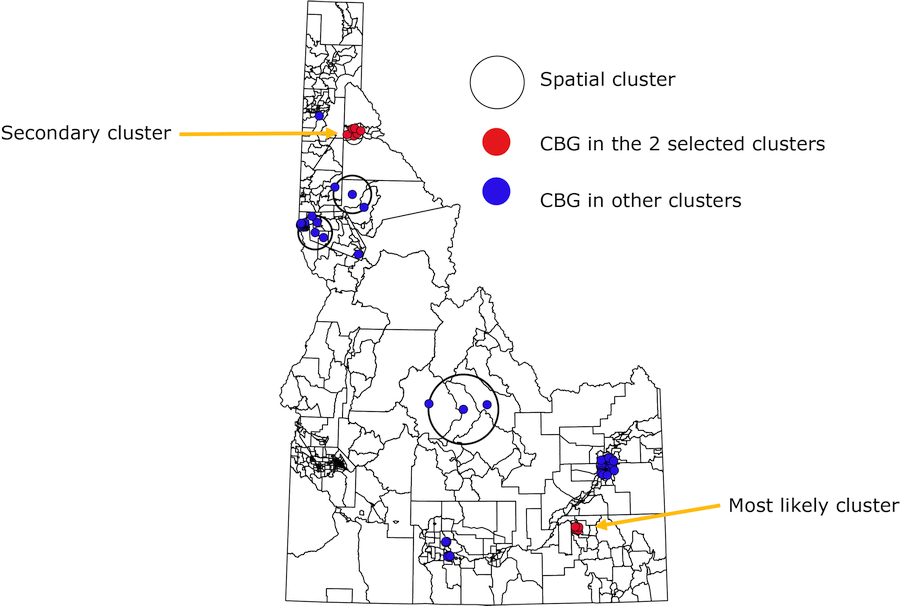
Spatial clusters of Idaho resident suicides by census block group, 2010–2014. A dot is a centroid of a census block group (CBG); 1 dot might represent 1 or more suicides that occurred in that CBG during the study period.

### Characteristics of census block groups in spatial clusters

Compared with census block groups outside spatial clusters of suicide, census block groups in spatial clusters were more likely to have a higher proportion of females, American Indians or Alaska Natives, never married persons, and persons in poverty, and a lower proportion of persons with less than a high school education (Table 3). Census block groups within spatial clusters had populations with a younger median age and a lower median household income. We observed no significant differences between census block groups within spatial clusters and outside spatial clusters in proportion Hispanic or Latino ethnicity, single-parent families, unemployment, renter-occupied housing, disability, or health insurance coverage. In the multivariable model that included significant characteristics from the univariable models, the following community characteristics remained significant: median age ≤31.1 years (multivariable-adjusted odds ratio [aOR] = 2.4; 95% confidence interval [CI], 1.04–5.6; *P* = .04), >53% female (aOR = 2.7; 95% CI, 1.3–5.8; *P* = .01), >1% American Indian or Alaska Native (aOR = 2.9; 95% CI, 1.4–6.3; *P* = .006), and >30% never married (aOR = 3.4; 95% CI, 1.5–8.0).

**Table 3:** Characteristics of Census Block Groups Within and Outside Spatial Clusters of Suicide, Idaho, 2010–2014^a^

Characteristic^b^	Census Block Groups Within Spatial Clusters Of Suicide, No. (%) (n = 31)	Census Block Groups Outside Spatial Clusters Of Suicide, No. (%) (n = 932)	Odds Ratio (95% Confidence Interval)^c^
**Proportion female**			
>0.53	14 (45.2)	226 (24.3)	2.6 (1.3–5.3)
≤0.53	17 (54.8)	706 (75.8)	1 [Reference]
**Median age**			
≤31.1 y	16 (51.6)	223 (23.9)	3.4 (1.7–7.0)
>31.1 y	15 (48.4)	709 (76.1)	1 [Reference]
**Proportion American Indian or Alaska Native**			
>0.01	15 (48.4)	225 (24.1)	3.0 (1.4–6.1)
≤0.01	16 (51.6)	707 (75.9)	1 [Reference]
**Proportion Hispanic or Latino**			
>0.16	5 (16.1)	235 (25.2)	0.6 (0.2–1.5)
≤0.16	26 (83.9)	697 (74.8)	1 [Reference]
**Proportion of never-married persons**			
>0.30	19 (61.3)	221 (23.7)	5.1 (2.4–10.7)
≤0.30	12 (38.7)	711 (76.3)	1 [Reference]
**Proportion of persons in single-parent families**			
>0.24	12 (38.7)	228 (24.5)	2.0 (0.9–4.1)
≤0.24	19 (61.3)	704 (75.5)	1 [Reference]
**Proportion of persons with <high school education^d^**			
>0.16	2 (6.5)	238 (25.5)	0.2 (0.1–0.9)
≤0.16	29 (93.6)	694 (74.5)	1 [Reference]
**Proportion of unemployed persons**			
>0.07	11 (35.5)	229 (24.6)	1.7 (0.8–3.6)
≤0.07	20 (64.5)	703 (75.4)	1 [Reference]
**Median household income, $**			
≤35,345	14 (45.2)	226 (24.3)	2.6 (1.3–5.3)
>35,345	17 (54.8)	706 (75.8)	1 [Reference]
**Proportion of persons in poverty**			
>0.22	13 (41.9)	227 (24.4)	2.2 (1.1–4.7)
≤0.22	18 (58.1)	705 (75.6)	1 [Reference]
**Proportion of persons in renter-occupied housing unit**			
>0.41	12 (38.7)	228 (24.5)	2.0 (0.9–4.1)
≤0.41	19 (61.3)	704 (75.5)	1 [Reference]
**Proportion of persons with disability**			
>0.22	8 (25.8)	232 (24.9)	1.1 (0.5–2.4)
≤0.22	23 (74.2)	700 (75.1)	1 [Reference]
**Proportion of persons with no health insurance coverage**			
>0.23	7 (22.6)	233 (25.0)	0.9 (0.4–2.1)
≤0.23	24 (77.4)	699 (75.0)	1 [Reference]

a Individual-level data on suicides obtained from death certificates stored by the Idaho Bureau of Vital Records and Health Statistics. Data on population estimates obtained from the 2010 US Census and data on community characteristics from the 2010–2014 American Community Survey’s 5-year estimates (25).

b Each variable for community characteristics was dichotomized into high and low levels. Except for age and income, we constructed the variables to compare the highest quartile with the lowest 3 quartiles for each variable. For age and income, we constructed the variables to compare the lowest quartile with the highest 3 quartiles for each variable.

c Based on Wald method from univariable logistic regression models.

d Did not receive a regular high school diploma, GED, or alternative credential.

## Discussion

This ecological study identified geographic areas with disproportionately high suicide rates at the census block group level in 2 parts of Idaho. The communities in areas with suicide clustering had a unique demographic and socioeconomic profile. To our knowledge, this is the first study to investigate spatial clustering of suicide in the western region of the United States.

The 2 spatial clusters of census block groups identified were in 2 of the 18 counties where high rates of suicide had been reported (21). Identifying these clusters provides a more detailed view of geographic areas in these counties: 25 census block groups in a county with 60 census block groups, and 6 census block groups in a county with 18 census block groups (21). Our findings on spatial clusters of suicide at the census block group level cannot be fully compared with findings from previous studies, because those studies used different geographic units (counties and census tracts, not census block groups) (15–20). The proportion of geographic units that were part of the identified clusters was smaller in Idaho (3%) than they were in Kentucky (13%) (15) and Florida (16%) (16). Despite different levels of geography with varying population compositions, this finding might be attributed to differences in suicide risk levels in each state; a state where suicide risk has less geographic variation (eg, Idaho) is less likely to have many clusters. Our study spanned 5 years, which is half of the study period of other US studies (15,16); a longer study including more suicides might have identified more or fewer areas or same or different areas within spatial clusters.

Our findings are generally consistent with findings of other studies reporting that areas of lower socioeconomic status are associated with higher rates of suicide (7). We found a positive association between suicide clustering and both low household income and high proportion of persons in poverty; however, we found a negative association between suicide clustering and low educational attainment. This finding is consistent with at least 1 previous study that found the proportion of the population without a diploma is less likely to be included in a suicide cluster (20). Our finding that suicide clustering was associated with a higher proportion of never-married persons is consistent with research on the influence of social support and family structure on suicide (8). Community characteristics related to housing, unemployment, disability, and health insurance coverage that were not significantly associated with suicide clustering in our study might be investigated in future studies to confirm our findings. Overall, the unique demographic and socioeconomic profile of areas with suicide clustering in Idaho should be viewed as a potential way to depict an environmental context that is conducive to suicide, rather than a direct cause of suicide clustering. 

The literature identified 2 possible explanations for suicide clustering. First, concentrations of persons at high risk for suicide might live in areas that could be identified as a cluster (compositional effects) (8). Second, place of residence might influence suicide risk by being less supportive (eg, because of social or economic isolation) of persons at high risk (contextual effect) (8). Our objective was not to investigate causation, and we did not incorporate individual-level data to assess individual risk of suicide after controlling for contextual effect.

Our study demonstrates the feasibility of a state health department investigation of spatial clusters of suicide using multiple data sources. Strengths of this study include the use of population-based suicide data; use of the census block group as a granular, detailed unit of geographic analysis; and consideration of a broad range of community characteristics that covered the same period as the suicides. Spatial cluster analysis using SaTScan has many advantages, including adjusting for population inhomogeneity, adjusting for multiple comparisons, adjusting for covariates, and limiting preselection bias by not specifying cluster size a priori (27). 

This study has several limitations. First, incorrectly not classifying suicide as a cause of death on death certification could have resulted in underreporting of suicide. Second, missing information on residential addresses resulted in incomplete geocoding; however, less than 2% of suicides were missing information on residential addresses. Third, we did not have information on how long the decedents lived in their homes; thus, we could not determine how duration of exposure to communities could affect results. Fourth, our cluster analysis was driven by the settings we selected in SaTScan; however, we followed the standard settings and those used in previous studies. Finally, our findings might not reflect current high-risk areas because data were from 2010–2014. However, retrospective analysis of mortality data is a fundamental tool for community health assessment, and we used the most recent available data. Although the contextual factors conducive to suicide in the identified clusters have probably not changed greatly since our study period, continuous evaluation and data triangulation to determine whether high-risk areas remain at high risk over time could increase confidence in public health programs that target prevention efforts to those areas. Although a study from Australia found that historical suicide clusters, detected during a 5-year period, predicted only 36% of suicide clusters detected during a subsequent 5-year period (29), our findings are better suited to inform current planning and response needs of suicide prevention programs rather than to predict future suicides.

Our findings could help public health practitioners and policy makers prioritize resources and target efforts for suicide prevention. The Centers for Disease Control and Prevention developed a technical package of prevention strategies to help communities use the best available evidence for suicide prevention (30). These strategies include strengthening economic supports; strengthening access and delivery of suicide care; creating protective environments; promoting connectedness; teaching coping and problem-solving skills; and identifying and supporting people at risk (30). A multicomponent public health suicide prevention approach should address the needs of communities at the highest risk of suicide, such as communities we found in our study. In Idaho, a public health approach that strengthens economic supports and strengthens access and delivery of suicide care in the identified areas might be most effective in preventing suicide.
